# Supplementary material for: LncRNA Ctcflos orchestrates transcription and alternative splicing in thermogenic adipogenesis
Source: EMBO Rep. 2021 May 31;22(7):e51289. doi: 10.15252/embr.202051289 (PMC8256291; doi:10.15252/embr.202051289)
Supplement: Supplementary file 1 — Expanded View Figures PDF [file EMBR-22-e51289-s003.pdf]

Expanded View Figures

Figure EV1. Regulation of protein-coding genes in brite adipogenesis, confirmation of *Ctcflos* regulation by qRT–PCR, Ctcflos KD effects on adipocyte lipid droplets, and mitochondrial biogenesis.

A Heatmap of positively and negatively regulated protein-coding genes selected according to correlation with *Ucp1*, regulation during differentiation and regulation by rosiglitazone treatment.

B, C Venn diagrams of positively and negatively regulated protein-coding genes selected according to correlation with *Ucp1*, regulation during differentiation and regulation by rosiglitazone treatment.

D *Ctcflos tr1* relative RNA levels in differentiated compared to undifferentiated primary brite adipocytes of 129S6 mice, assessed by qPCR. Mean and individual values, $n = 3$ (biological replicates), unpaired t-test, ***$P < 0.001$.

E *Ctcflos tr1* relative RNA levels in differentiated brite (+rosi) compared with differentiated white (−rosi) adipocytes of 129S6 mice, assessed by qPCR. Mean and individual values, $n = 3$ (biological replicates), unpaired t-test, *$P < 0.05$.

F Correlation of *Ctcflos* and *Ucp1* transcript levels in iBAT of C57BL/6J mice across 0, 6, 24, and 48 h of cold (4°C) exposure (transcript levels in RPKM). Individual values. Pearson correlation, ***$P < 0.001$.

G Protein-coding potential scores for *Ctcflos*, *Nuclear Paraspeckle Assembly Transcript 1* (*Neat1*), *Myoregulin* (*Mrln*), and *Ucp1* assessed by publicly available machine-learning algorithms.

H *In silico* translation of *Ctcflos* transcripts and alignment against the NCBI protein database to test for the putative presence of corresponding peptides.

I Relative *Ucp1* pre-mRNA levels in response to *Ctcflos* tr1 KD by ASO 1 compared with nontargeting control, assessed by qPCR. Mean and individual values, $n = 2$–3 (biological replicates).

J Number of lipid droplets in *Ctcflos* tr1 (ASO 1) KD compared with control cells, assessed by digital image analysis (Wimasis). Mean values ± SD, $n = 3$ (biological replicates), unpaired t-test, *$P < 0.05$.

K–M Lipid droplet morphology in *Ctcflos* tr1 KD compared with controls. (K) Lipid droplet size distribution as percentage of lipid droplets in different size categories, assessed by digital image analysis (Wimasis), $n = 3$ (biological replicates). (L) Microscopic image section of differentiating primary brite adipocytes 72 h after *Ctcflos* tr1 KD (lower image) or nontargeting control treatment (upper image) (day 1 diff). (M) Percentage of lipid droplets below and above 40 μm cutoff comparing *Ctcflos* tr1 KD and control samples. Mean values ± SD, $n = 3$ (biological replicates), two-way ANOVA (Šídák-test).

N, O Oil red O staining of control and *Ctcflos* tr1 (ASO 1) KD primary brite adipocytes 72 h after the KD at day 1 of differentiation. (N) Images of entire wells (left) of stained cells, microscopic images with 10× objective, bars indicate 200 μm (middle) and microscopic images with 32× objective, bars indicate 100 μm (right). (O) Quantification of Oil red O staining by absorbance measurement at 492 nm of stained cells subtracted by background. Mean and individual values, $n = 3$ (technical replicates), unpaired t-test, *$P < 0.05$.

P Quantification of Mito Tracker staining as whole image fluorescence intensity subtracted by background and normalized to cell number comparing *Ctcflos* tr1 (ASO 1) KD and control. Mean and individual values $n = 6$–7 (technical replicates) the experiment was repeated a second time for a total of two biological replicates, for the second replication see (Fig 4S), unpaired t-test, **$P < 0.01$.

© 2021 The Authors

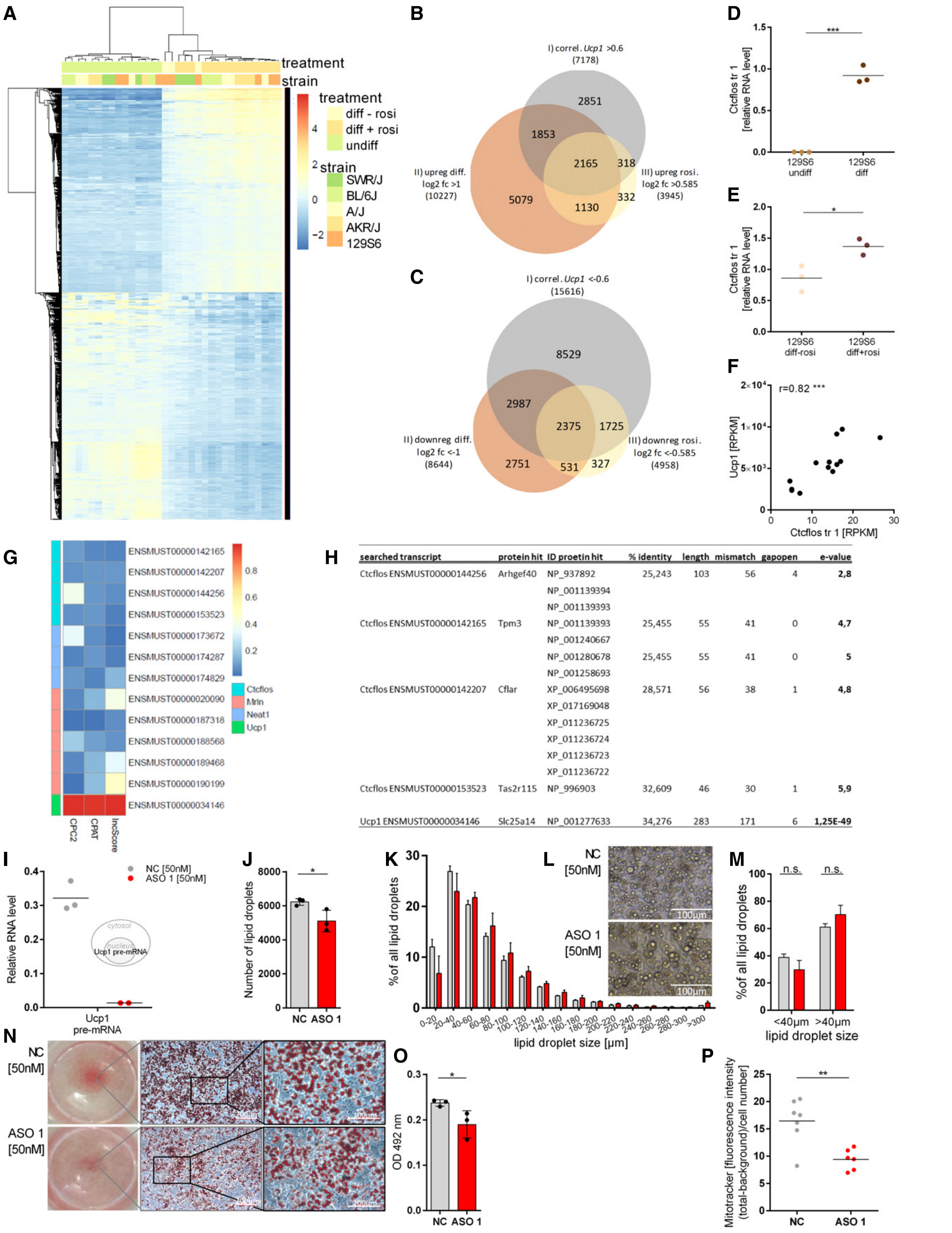

Figure EV1.

 © 2021 The Authors

Figure EV2. *Ctcflos* KD effects on inguinal preadipocytes, mature brite adipocytes, and differentiating brown and white adipocytes.

A Design of *Ctcflos* knockdown (KD) experiments in inguinal preadipocytes and mature brite adipocytes. For experiments in preadipocytes, *Ctcflos* KD was performed at sub-confluence of growing precursor cells and cells were harvested and analyzed 72 h later. For experiments in mature brite adipocytes, *Ctcflos* KD was performed at day 5 of differentiation and cells were harvested and analyzed 72 h later.

B Relative *Ucp1* transcript levels in response to *Ctcflos* tr1 (ASO 1) KD in sub-confluent preadipocytes compared with nontargeting control, assessed by qPCR. Mean values ± SD, $n = 3$ (technical replicates), unpaired t-test, *$P < 0.05$.

C–I Impact of *Ctcflos* KD on mature brite adipocytes. (C, D) Efficiency of *Ctcflos* KD by (C) LNA Gapmer ASO 1 targeting *Ctcflos* transcript 1 and (D) LNA Gapmer ASO 2 targeting *Ctcflos* transcripts 3 and 4 compared with nontargeting controls, assessed by qPCR. Mean values ± SD, $n = 3$ (biological replicates), unpaired t-tests, **$P < 0.01$, ***$P < 0.001$. (E) Relative *Ucp1* transcript levels in response to *Ctcflos* tr1 (ASO 1), 3, and 4 (ASO 2) KD compared with nontargeting controls, assessed by qPCR. Mean values ± SD, $n = 3$ (biological replicates), unpaired t-tests, **$P < 0.01$. (F) Relative expression levels of *cell death-inducing DNA fragmentation factor alpha-like effector A* (*Cidea*) and *cytochrome c oxidase subunit 7a1* (*Cox7a1*) in *Ctcflos* tr1 (ASO 1), 3, and 4 (ASO 2) KD compared with control samples, assessed by qPCR. Mean and individual values, $n = 2–3$ (biological replicates), for groups with $n = 3$ (biological replicates): unpaired t-tests or in gray parenthesis two-way ANOVA (Šídák-test), *$P < 0.05$, **$P < 0.01$, ***$P < 0.001$. (G-I) Effect of *Ctcflos* KD on mature brite adipocyte respiratory capacity. (G) Time course of oxygen consumption rates in mature primary brite adipocytes 72 h after treatment (day 5 diff), comparing *Ctcflos* tr1 (ASO 1), 3, and 4 (ASO 2) KD with their respective controls, measured by microplate-based respirometry (Seahorse XF96 Analyzer). Oxygen consumption is recorded under basal conditions and in response to successive injection of oligomycin (5 μM), isoproterenol (1.5 μM), FCCP (1 μM) and antimycin A (5 μM) to determine basal leak, UCP1-dependent uncoupled, maximal, and non-mitochondrial respiration, respectively. Data are expressed after deduction of non-mitochondrial respiration. Mean values ± SD, $n = 4$ (biological replicates). (H, I) Quantification of isoproterenol-stimulated UCP1-mediated uncoupled respiration, expressed as fold of basal leak respiration. Mean and single values, paired t-test, *$P < 0.05$, **$P < 0.01$.

J Design of *Ctcflos* knockdown (KD) experiments in differentiating interscapular brown and inguinal white adipocytes. *Ctcflos* KD was performed at the first day after induction, and cells were harvested and analyzed 72 h later.

K–N Impact of *Ctcflos* KD on differentiating interscapular brown adipocytes. (K) Relative *Ucp1* transcript levels in response to *Ctcflos* tr1 (ASO 1), 3, and 4 (ASO 2) KD (day 1 diff) compared with nontargeting controls, assessed by qPCR. Mean values ± SD, $n = 3–4$ (biological replicates), unpaired t-tests, n.s. $P > 0.05$, **$P < 0.01$. (L, M) Effect of *Ctcflos* KD on differentiating brown adipocyte respiratory capacity. (L) Time course of oxygen consumption rates in differentiating primary brown adipocytes 72 h after treatment (day 1 diff), comparing *Ctcflos* tr1 (ASO 1), 3, and 4 (ASO 2) KD with their respective controls, measured by microplate-based respirometry (Seahorse XF96 Analyzer) as described above for (G). Mean values, $n = 8$ (technical replicates). (M, N) Quantification of isoproterenol-stimulated UCP1-mediated uncoupled respiration, expressed as fold of basal leak respiration. Mean and single values, paired t-test, **$P < 0.01$.

O Impact of *Ctcflos* KD on differentiating white adipocytes. Relative expression levels of *Adipose triglyceride lipase* (*Atgl*) and *Hormone-sensitive lipase* (*Hsl*), *Fatty acid-binding protein 4 (Fabp4)*, and *Leptin* in *Ctcflos* tr1 (ASO 1), 3, and 4 (ASO 2) KD (day 1 diff) compared with control samples, assessed by qPCR. Mean values ± SD, $n = 3$ (biological replicates), unpaired t-tests, or in gray parenthesis two-way ANOVA (Šídák-test), *$P < 0.05$, **$P < 0.01$.

P, Q Role of *Ctcfl* in *Ctcflos*-dependent brite adipogenesis. (P) Relative *Ctcfl* transcript levels in response to *Ctcflos* tr1 (ASO 1) KD (day 1 diff) compared with nontargeting controls, assessed by qPCR. Mean values ± SD, $n = 3$ (biological replicates), unpaired t-test, n.s. $P > 0.05$. (Q) Relative *Ctcfl* transcript levels in response to *Ctcfl* KD (dsi Ctcfl) (day 1 diff) compared with nontargeting controls and relative *Ucp1* transcript levels in response to *Ctcfl* KD (dsi Ctcfl) (day 1 diff) compared with nontargeting controls, assessed by qPCR. Mean values ± SD, $n = 3$ (biological replicates). Unpaired t-tests or in gray parenthesis two-way ANOVA (Šídák-test), n.s. $P > 0.05$, *$P < 0.05$.

R, S Role of Pck1 in *Ctcflos*-dependent brite adipogenesis. (R) Relative *Pck1* transcript levels in response to *Ctcflos* tr1 (ASO 1) KD (day 1 diff) compared with nontargeting controls, assessed by qPCR. Mean values ± SD, $n = 3$ (technical replicates), unpaired t-test, ***$P < 0.001$. (S) Relative *Pck1* transcript levels in response to *Pck1* KD (si Pck1) (day 1 diff) compared with nontargeting controls and relative *Ucp1* transcript levels in response to *Pck1* KD (si Pck1) (day 1 diff) compared with nontargeting controls, assessed by qPCR. Mean values ± SD, $n = 6$ (technical replicates). Unpaired t-tests, or in gray parenthesis two-way ANOVA (Šídák-test), n.s. $P > 0.05$, ****$P < 0.0001$.

© 2021 The Authors

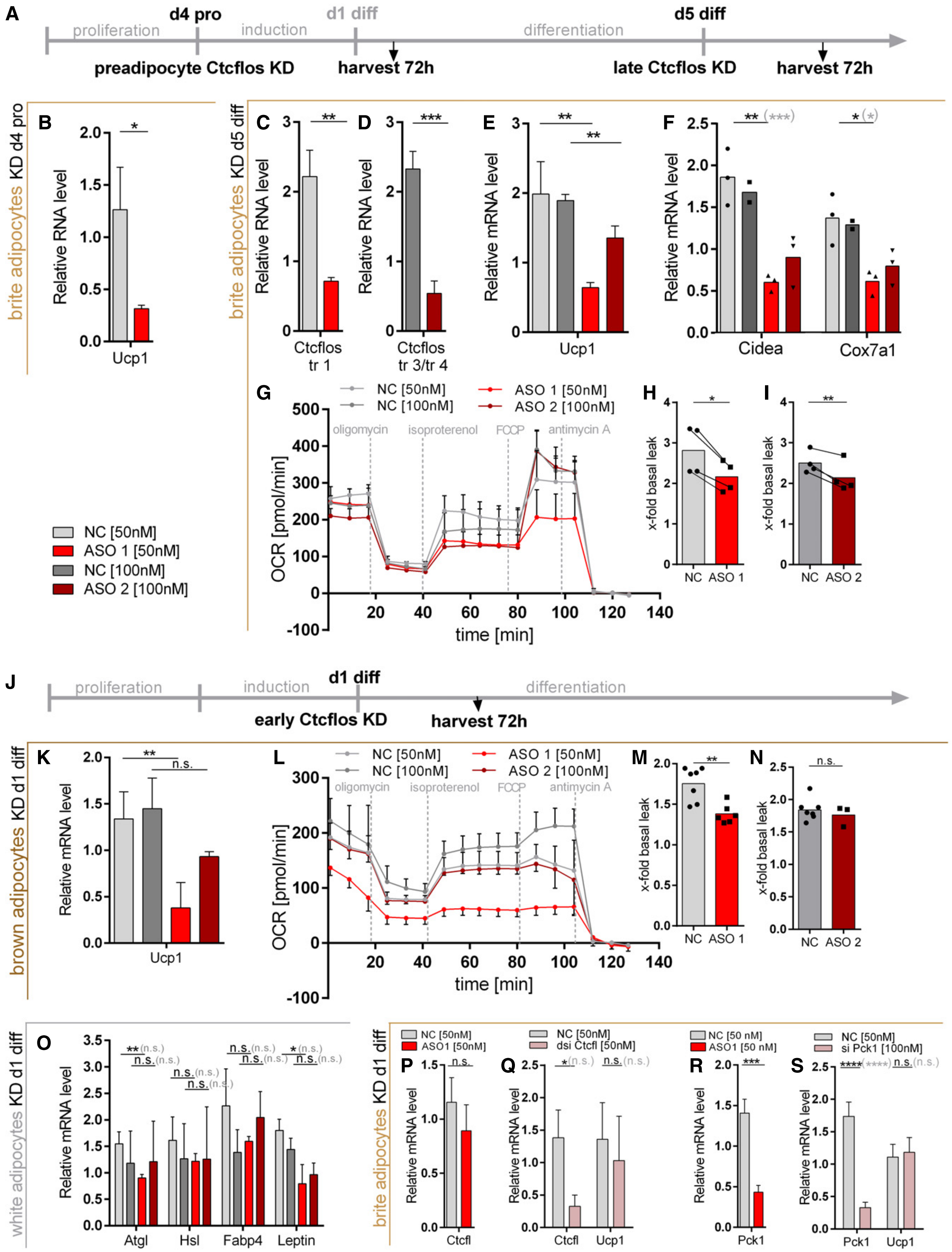

Figure EV2.

 © 2021 The Authors

Figure EV3. Impact of *Ctcflos* KD on mitochondrial respiratory chain, brite adipogenesis, and general adipogenesis in differentiating and mature brite cells in transcriptome profiling. Epistasis between *Ctcflos* and *Prdm16*.

A–C Impact of *Ctcflos* KD on differentiating brite adipocytes (KD on day 1 of differentiation) in transcriptome analysis.(A) Expression of respiratory chain complex subunits in response to *Ctcflos* tr1 (ASO1) KD (day 1 of differentiation), visualized by Path Visio software. Color code visualizes log2 fold change of gene expression 72 h after the KD. (B) Relative expression levels of selected respiratory chain complex subunits. *Ctcflos* tr1 (ASO 1) and tr3 and tr4 (ASO2) KD samples relative to their nontargeting controls. *NADH:ubiquinone oxidoreductase subunit A3* (*Ndufa3*), *NADH:ubiquinone oxidoreductase core subunit S4* (*Ndufs4*), *succinate dehydrogenase complex, subunit B, iron sulfur* (*Sdhb*), *ubiquinol-cytochrome c reductase, complex III subunit VII* (*Uqcrq*), *cytochrome c oxidase subunit 5B* (*Cox8b*), *ATP synthase subunit E* (*Atp5k*), *ATP synthase subunit s* (*Atp5s*). Mean values ± SD, $n = 6$ (biological replicates), unpaired *t*-tests or in gray parenthesis two-way ANOVA (Šídák-test), *$P < 0.05$, **$P < 0.01$, ***$P < 0.001$, ****$P < 0.0001$, no * indicate nonsignificant changes (not depicted in the graph due to visibility reasons). (C) Relative expression levels of general adipogenesis marker genes. *Ctcflos* tr1 (ASO 1) and tr3 and tr4 (ASO2) KD samples relative to their nontargeting controls. *Adipose triglyceride lipase* (*Atgl*), *hormone-sensitive lipase* (*Hsl*), *Adiponectin* (*AdipoQ*), *fatty acid-binding protein 4* (*Fabp4*), *Peroxisome proliferator-activated receptor alpha* (*Ppara*), *sterol regulatory element-binding transcription factor 1* (*Srebf1*), *CCAAT/enhancer-binding protein alpha* (*Cebpa*). Mean values ± SD, $n = 6$ (biological replicates), unpaired *t*-tests or in gray parenthesis two-way ANOVA (Šídák-test), *$P < 0.05$, **$P < 0.01$,, ****$P < 0.0001$, no * indicate nonsignificant changes (not depicted in the graph due to visibility reasons).

D, E Impact of *Ctcflos* KD on mature brite adipocytes (KD on day 5 of differentiation) in transcriptome analysis. (D) Relative expression levels of brite adipocyte marker genes. *Ctcflos* tr1 (ASO 1), tr3 and tr4 (ASO2) KD samples relative to their nontargeting controls. *Uncoupling protein 1* (*Ucp1*), *cell death-inducing DNA fragmentation factor alpha-like effector A* (*Cidea*), *cytochrome c oxidase subunit 7a1* (*Cox7a1*), *peroxisome proliferator-activated receptor γ coactivator 1α* (*Pgc1a*), and *elongation of very long chain fatty acid-like 3* (*Elovl3*). Mean values ± SD, $n = 3$ (biological replicates), unpaired *t*-tests or in gray parenthesis two-way ANOVA (Šídák-test), *$P < 0.05$, **$P < 0.01$, no * indicate nonsignificant changes (not depicted in the graph due to visibility reasons). (E) Relative expression levels of general adipogenesis marker genes. *Ctcflos* tr1 (ASO 1), tr3, and tr4 (ASO2) KD samples relative to their nontargeting controls. *Atgl*, *Hsl*, *AdipoQ*, *Fabp4*, *Ppara*, *Srebf1*, and *Cebpa*. Mean values ± SD, $n = 3$ (biological replicates), unpaired *t*-tests or in gray parenthesis two-way ANOVA (Šídák-test), *$P < 0.05$, **$P < 0.01$, ***$P < 0.001$, no * indicate nonsignificant changes (not depicted in the graph due to visibility reasons).

F–I Epistasis between *Ctcflos* and *Prdm16*. (F) Paired TOST equivalence test comparing *Ucp1* transcript levels of (A) *Ctcflos* single with *Ctcflos*+*Prdm16* double KD. Significance level alpha = 0.1. Equivalence bounds based on the multiplicative gene interaction model. (G-I) Rescue of *Ctcflos* KD impact on *Ucp1* gene transcription by *Prdm16* overexpression. Primary iWAT cells untreated or infected with Prdm16-expressing viral particles at the second day of proliferation, followed by reverse transfection at the first day of differentiation using nontargeting control (NC) or *Ctcflos*-targeting ASO1. Gene expression analyzed 72 h later by qPCR. (G) Transcript levels of *Ctcflos* tr1 relative to *Gtf2b*. (H) Transcript levels of *Prdm16* relative to *Gtf2b*. (I) Transcript levels of *Ucp1* relative to *Gtf2b*. Mean and individual values. Each graph presents pooled data from two experiments with slightly varying virus titers. RM One-way ANOVA (Tukey test), n.s. $P > 0.05$, *$P < 0.05$, **$P < 0.01$.

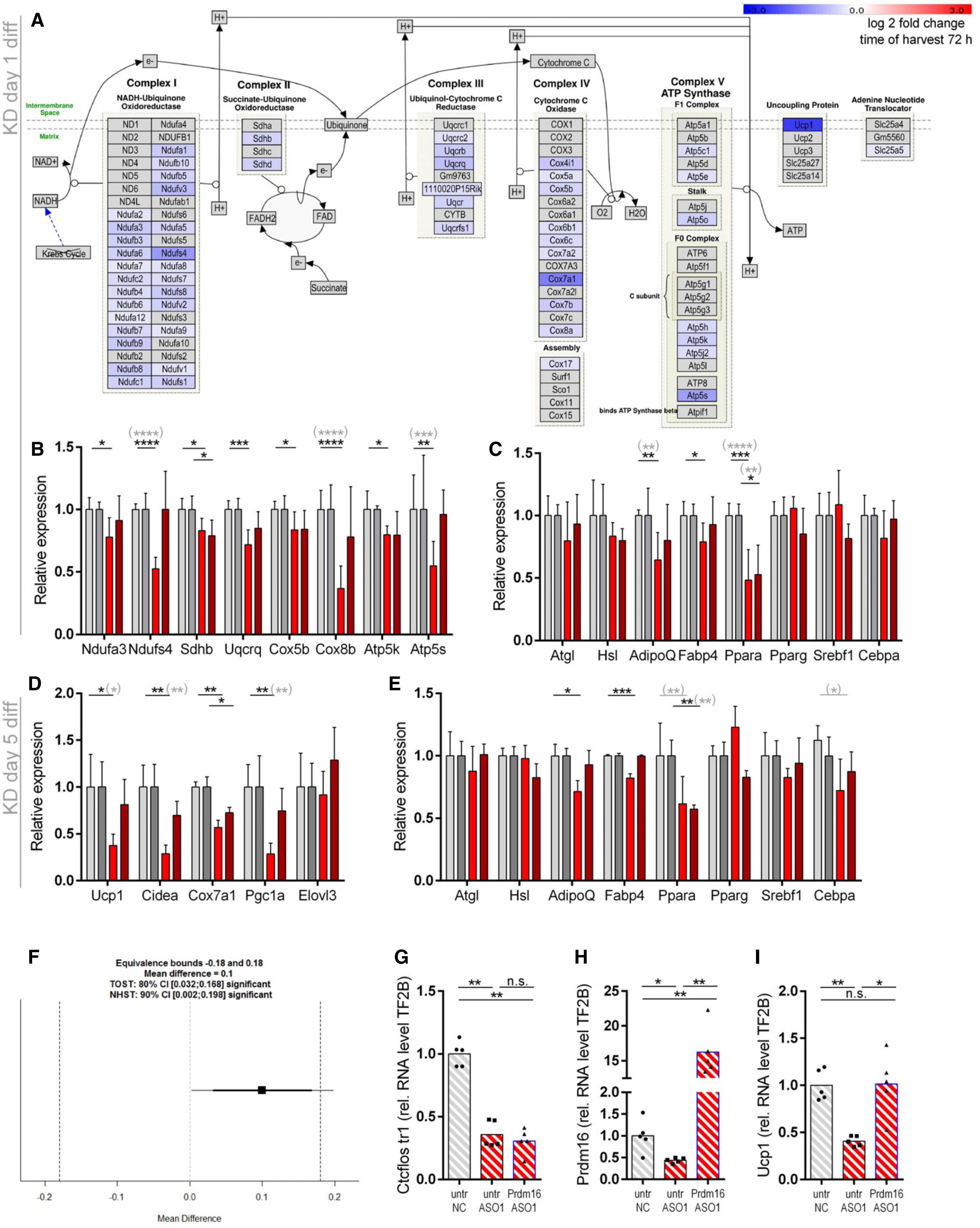
KD day 1 diff
A
Complex I
NADH-Ubiquinone Oxidoreductase
Complex II
Succinate-Ubiquinone Oxidoreductase
Complex III
Ubiquinol-Cytochrome C Reductase
Complex IV
Cytochrome C Oxidase
Complex V
ATP Synthase
Uncoupling Protein
Adenine Nucleotide Translocator
log 2 fold change
time of harvest 72 h
B
C
Relative expression
Ndufa3 Ndufs4 Sdhb Uqcrq Cox5b Cox8b Atp5k Atp5s
Atgl Hsl AdipoQ Fabp4 Ppara Pparg Srebf1 Cebpa
KD day 5 diff
D
E
Ucp1 Cidea Cox7a1 Pgc1a Elovl3
F
Equivalence bounds -0.18 and 0.18
Mean difference = 0.1
TOST: 80% CI [0.032;0.168] significant
NHST: 90% CI [0.002;0.198] significant
Mean Difference
G
H
I
Ctcflos tr1 (rel. RNA level TF2B)
Prdm16 (rel. RNA level TF2B)
Ucp1 (rel. RNA level TF2B)
untr NC
untr ASO1
Prdm16 ASO1
n.s.

Figure EV3.

Figure EV4. *Ctcflos* KD-dependent changes in the splicing machinery.

A Expression of splicing machinery components and splicing regulating factors from transcriptome profiling in response to *Ctcflos* tr1 (ASO 1) KD (KD on day 1 of differentiation), visualized by Path Visio software. Color code visualizes log2 fold change in gene expression 24 h after the KD.

B, C Quantification of SC35 fluorescence signal from immunocytochemistry as whole image fluorescence intensity subtracted by background and normalized to total cell number. Mean and individual values, $n = 8–11$ (technical replicates) the experiment was performed for a total of three times, for the third experiment see (Fig 6F), unpaired t-test, $*P < 0.05$, $**P < 0.01$.

D, E Quantification of nuclear speckle number from immunocytochemistry as mean number of SC35 signals per nucleus. Mean and individual values, $n = 8–11$ (technical replicates) the experiment was performed for a total of three times, for the third experiment see (Fig 6G), unpaired t-test, $**P < 0.01$.

F–Q Correlation of SC35 and UCP1 fluorescence signals in immunocytochemistry across individual cells in (F-H, L-N) control and (I-K, O-Q) *Ctcflos* tr1 (ASO 1) KD cells. (F-K) Extreme values are included. (L-Q) Extreme values were excluded to avoid distortion of correlation analysis. Additional three replicates to (Fig 6F and G). Pearson correlation, $*P < 0.05$, $**P < 0.01$, $****P < 0.0001$.

© 2021 The Authors

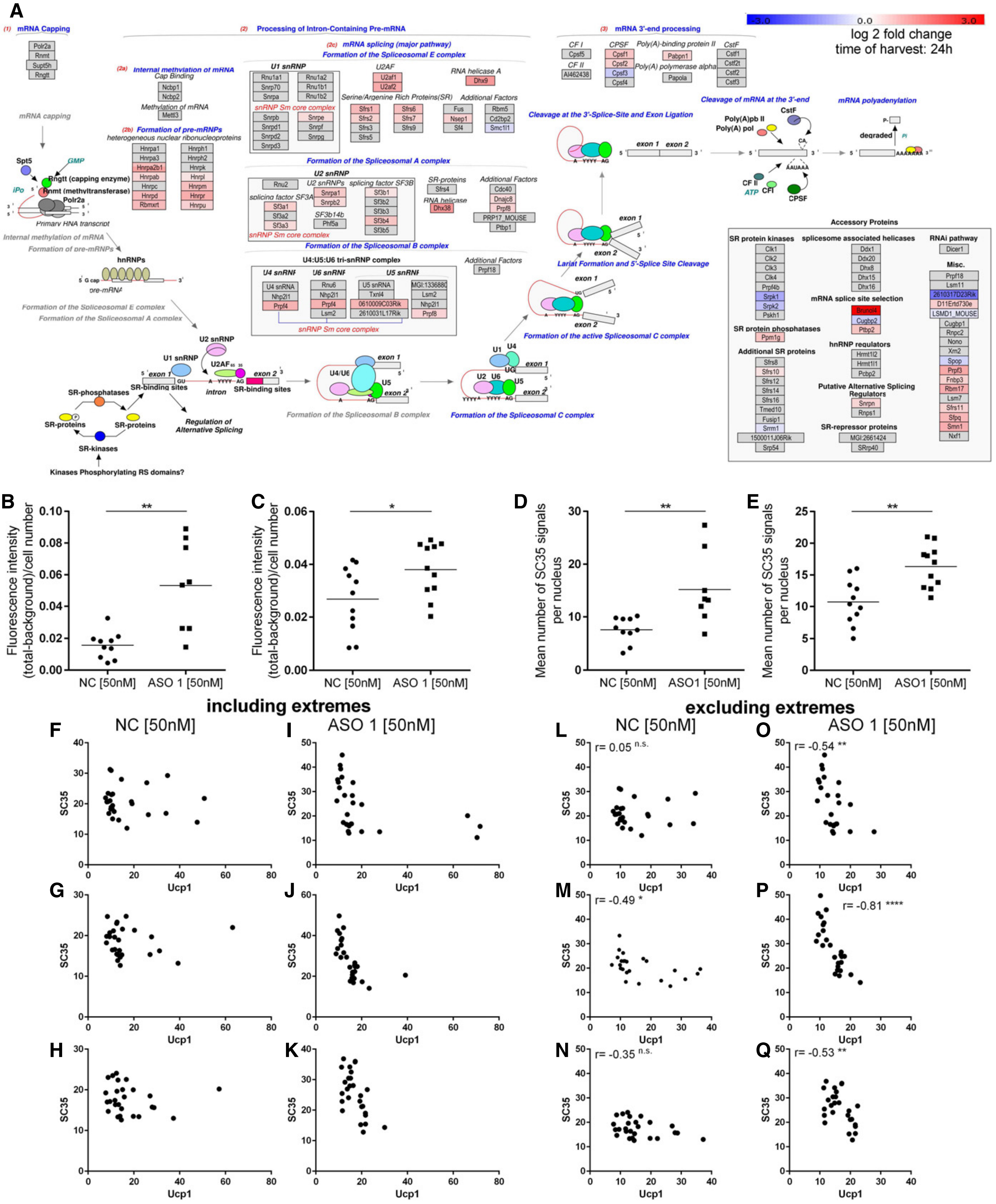
A
log 2 fold change
time of harvest: 24h
-3.0
0.0
3.0
(1) mRNA Capping
(2) Processing of Intron-Containing Pre-mRNA
(3) mRNA 3'-end processing
(2a) Internal methylation of mRNA
(2b) Formation of pre-mRNPs
(2c) mRNA splicing (major pathway)
Formation of the Spliceosomal E complex
Formation of the Spliceosomal A complex
Formation of the Spliceosomal B complex
Formation of the Spliceosomal C complex
Formation of the active Spliceosomal C complex
Lariat Formation and 5'-Splice Site Cleavage
Cleavage at the 3'-Splice-Site and Exon Ligation
Cleavage of mRNA at the 3'-end
mRNA polyadenylation
Accessory Proteins
SR protein kinases
spliceosome associated helicases
RNAi pathway
mRNA splice site selection
SR protein phosphatases
hnRNP regulators
Additional SR proteins
Putative Alternative Splicing Regulators
SR-repressor proteins
Misc.
Kinases Phosphorylating RS domains?
Regulation of Alternative Splicing
B
C
D
E
Fluorescence intensity (total-background)/cell number
Mean number of SC35 signals per nucleus
NC [50nM]
ASO 1 [50nM]
ASO1 [50nM]
**
*
including extremes
excluding extremes
F
G
H
I
J
K
L
M
N
O
P
Q
SC35
Ucp1
r= 0.05 n.s.
r= -0.54 **
r= -0.49 *
r= -0.81 ****
r= -0.35 n.s.
r= -0.53 **

Figure EV4.

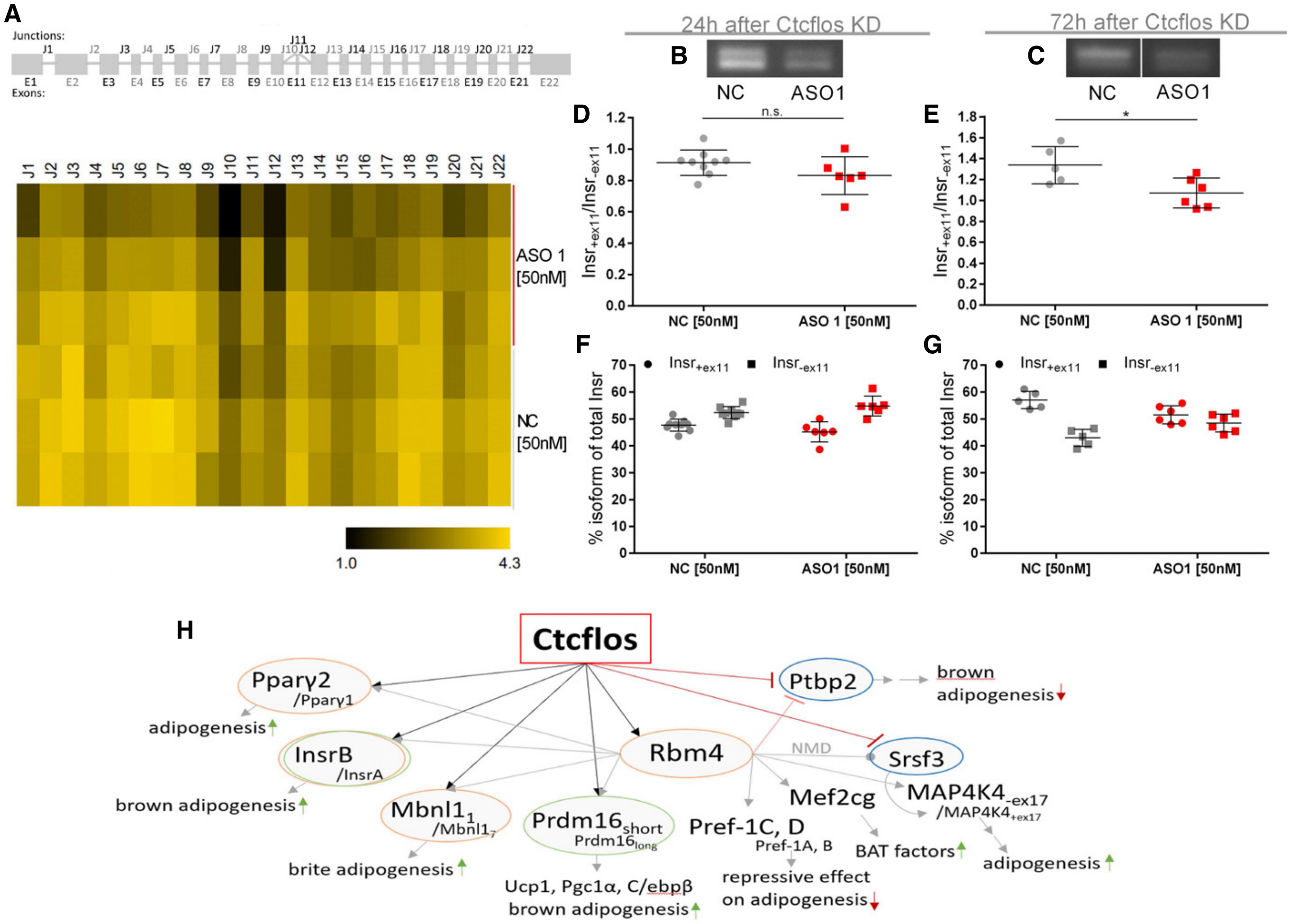

Figure EV5. *Ctcflos* KD-dependent changes in alternative splicing of *Insr*.

A Splice graph (upper panel) and exon junction heatmap (lower panel) of *Insulin receptor (Insr)* comparing *Ctcflos* tr1 (ASO 1) KD and controls in transcriptome wide splicing analysis by SGSeq algorithm (Goldstein *et al*, 2016). The splice graph displays *Insr* exons and alternative exon junctions. In the heatmap, usage of exon junctions is quantified from junction spanning reads for *Ctcflos* tr1 (ASO 1) KD and controls.

B, C PCR gel pictures of $Insr_{+ex11}$ (upper band) and $Insr_{-ex11}$ (lower band) in control and *Ctcflos* tr1 (ASO 1) KD samples, (B) 24 h or (C) 72 h after the KD.

D, E Ratio of $Insr_{+ex11}$ to $Insr_{-ex11}$ in control and *Ctcflos* tr1 (ASO 1) KD samples, (D) 24 h or (E) 72 h after the KD quantified from PCR signals. Mean and individual values, $n = 5–9$ (technical replicates), unpaired *t*-test, n.s. $P > 0.05$ $*P < 0.05$.

F, G Percentage of $Insr_{+ex11}$ and $Insr_{-ex11}$ of total *Insr* in control and *Ctcflos* tr1 (ASO 1) KD samples, (F) 24 h or (G) 72 h after the KD quantified from PCR signals. Mean and individual values, $n = 5–9$ (technical replicates).

H Reported targets of RBM4 regulated alternative splicing and transcription in brown adipose tissue (Lin *et al*, 2014; Lin, 2015; Chi & Lin, 2018; Peng *et al*, 2018; Hung & Lin, 2019). Yellow circles mark genes that were identified by deep sequencing to be differentially spliced also by *Ctcflos* tr1 (ASO 1) KD compared with control samples, green circles mark genes for which *Ctcflos*-dependent alternative splicing was confirmed experimentally, and blue circles highlight genes that are transcriptionally regulated by *Ctcflos*.

Source data are available online for this figure.
