# Supplementary figures and images for: LncRNA Ctcflos orchestrates transcription and alternative splicing in thermogenic adipogenesis
Source: EMBO Rep. 2021 May 31;22(7):e51289. doi: 10.15252/embr.202051289 (PMC8256291; doi:10.15252/embr.202051289)

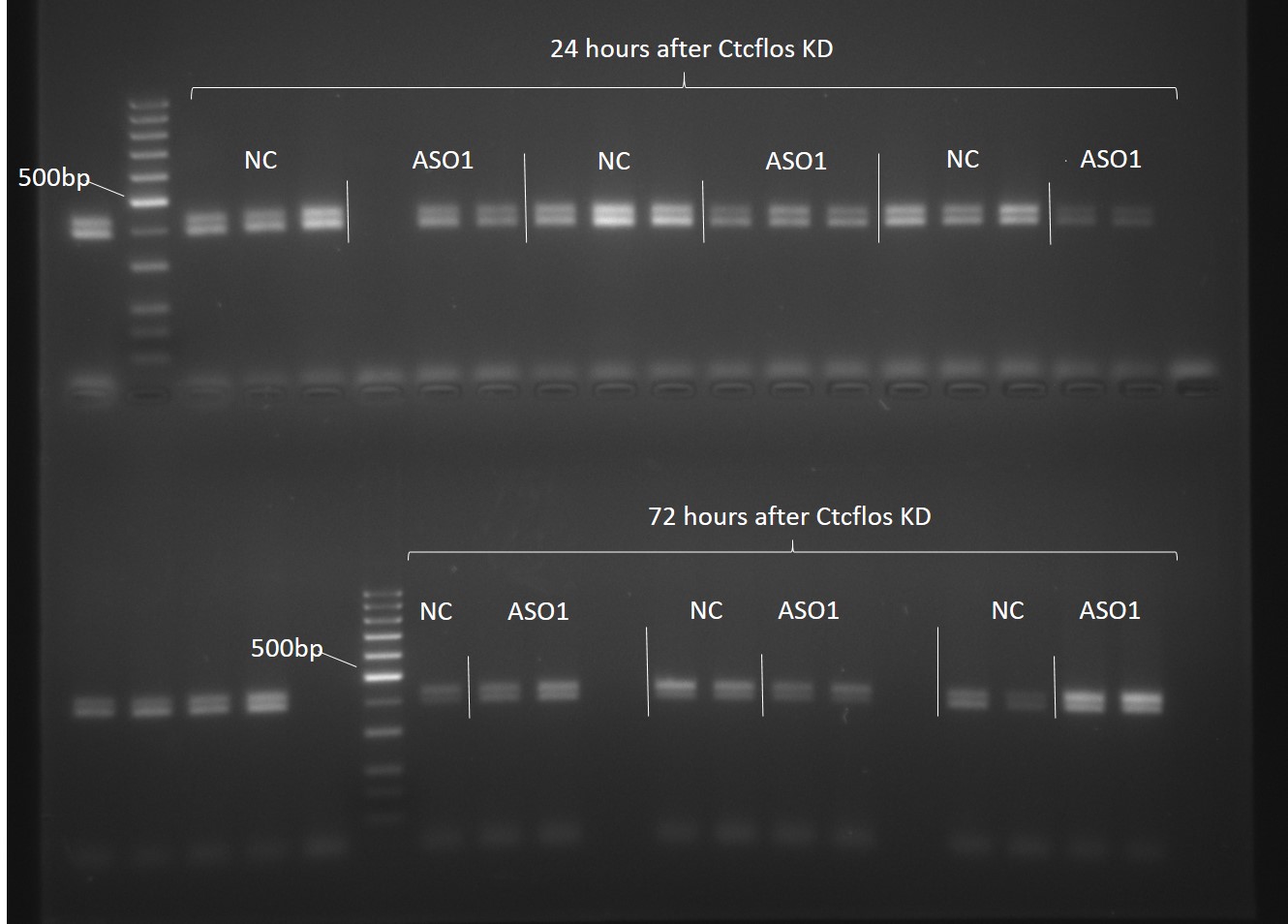

Supplement: Supplementary file 3 — Source Data for Expanded View [file EMBR-22-e51289-s001.zip › embr202051289-sup-0005-SDataFigEV5.jpg]

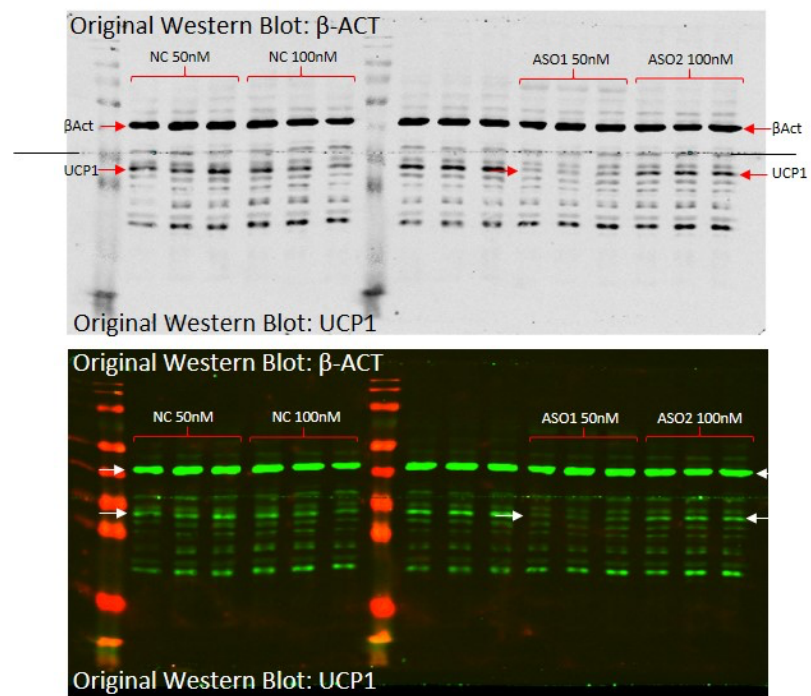

Figure 4G

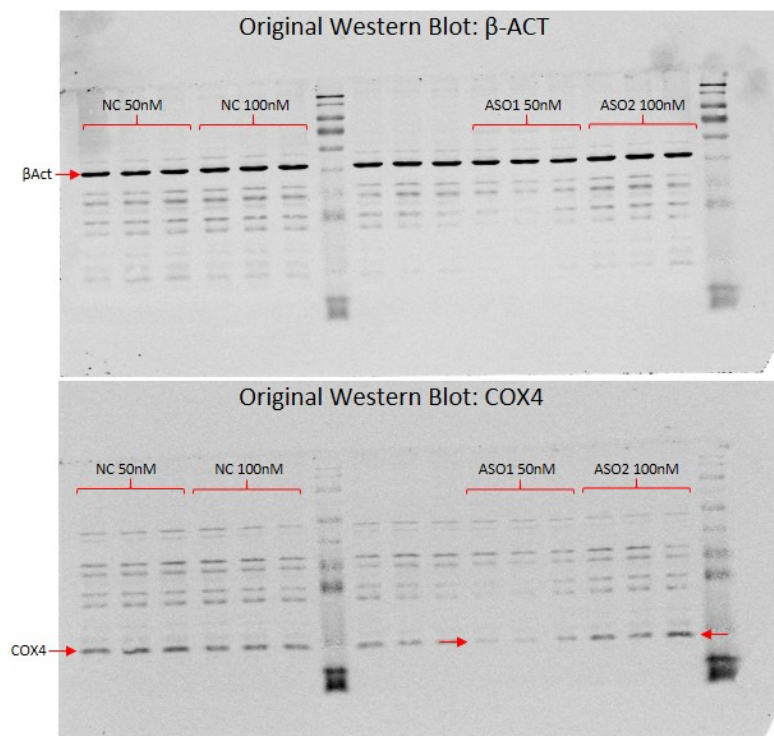

Figure 4Q

Supplement: Supplementary file 5 — Source Data for Figure 4 [file EMBR-22-e51289-s006.pdf]

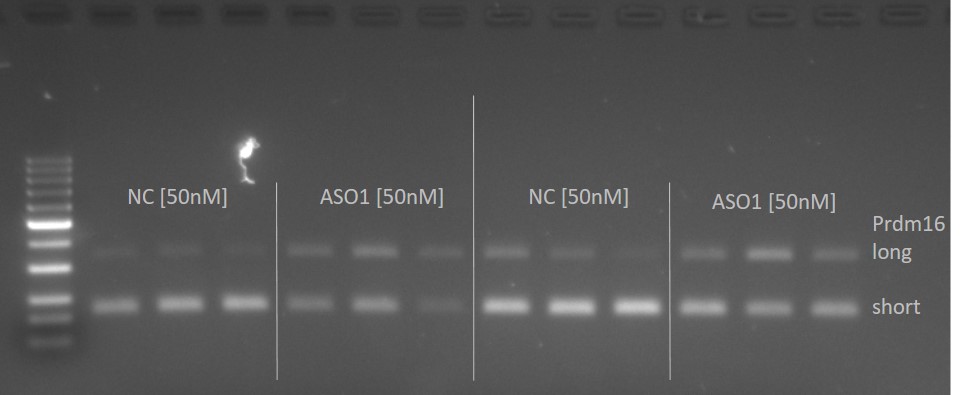

Supplement: Supplementary file 6 — Source Data for Figure 6 [file EMBR-22-e51289-s004.jpg]
